# Exposure Route of TiO_2_ NPs from Industrial Applications to Wastewater Treatment and Their Impacts on the Agro-Environment

**DOI:** 10.3390/nano10081469

**Published:** 2020-07-27

**Authors:** Zahra Zahra, Zunaira Habib, Sujin Chung, Mohsin Ali Badshah

**Affiliations:** 1 Department of Civil & Environmental Engineering, University of California-Irvine, Irvine, CA 92697, USA; 2 Institute of Environmental Sciences and Engineering, School of Civil and Environmental Engineering, National University of Sciences and Technology, Islamabad 44000, Pakistan; zhabib.phdiese@student.nust.edu.pk; 3 Plamica Labs, Batten Hall, 125 Western Ave, Allston, MA 02163, USA; thesujins@gmail.com; 4 Department of Chemical and Biomolecular Engineering, University of California-Irvine, Irvine, CA 92697, USA; badshahm@uci.edu

**Keywords:** TiO_2_ NPs, applications, wastewater treatment, agro-environment, exposure pathways

## Abstract

The tremendous increase in the production and consumption of titanium dioxide (TiO_2_) nanoparticles (NPs) in numerous industrial products and applications has augmented the need to understand their role in wastewater treatment technologies. Likewise, the deleterious effects of wastewater on the environment and natural resources have compelled researchers to find out most suitable, economical and environment friendly approaches for its treatment. In this context, the use of TiO_2_ NPs as the representative of photocatalytic technology for industrial wastewater treatment is coming to the horizon. For centuries, the use of industrial wastewater to feed agriculture land has been a common practice across the globe and the sewage sludge generated from wastewater treatment plants is also used as fertilizer in agricultural soils. Therefore, it is necessary to be aware of possible exposure pathways of these NPs, especially in the perspective of wastewater treatment and their impacts on the agro-environment. This review highlights the potential exposure route of TiO_2_ NPs from industrial applications to wastewater treatment and its impacts on the agro-environment. Key elements of the review present the recent developments of TiO_2_ NPs in two main sectors including wastewater treatment and the agro-environment along with their potential exposure pathways. Furthermore, the direct exposure routes of these NPs from production to end-user consumption until their end phase needs to be studied in detail and optimization of their suitable applications and controlled use to ensure environmental safety.

## 1. Introduction

Nanotechnology has touched every field by its scientific novelties. Although the use of nanotechnology is at the early stage, it appears to have significant effects in different areas. It offers great potential for the use of nanomaterials (NMs) in various fields related to all public and industrial sectors, including material, energy, agriculture, healthcare, communication, and information technologies. NMs are the materials that have at least one dimension on the nanoscale [1]. Titanium dioxide with formula TiO_2_ is the most important binary metal oxide material which exists in three naturally occurring solid phases; anatase (3.2 eV), rutile (3.0 eV), and brookite (3.2 eV) [2]. Anatase is mostly used in photocatalysis and recognized as a major phase of commercial TiO_2_. Degussa P25 is widely used commercial TiO_2_ nanoparticles (NPs) with an anatase to rutile phase ratio of 4:1. It has always been a hot topic to attain high crystalline TiO_2_ NPs with tunable functions by manipulating its morphology. In this context, mesocrystal TiO_2_ has gained much attention with remarkable photocatalytic activity in several applications [3]. Moreover, TiO_2_ NPs have unique characteristics of a very high refractive index, whiteness, and opacity, efficiency, increased chemical stability, and minimum cost [4]. TiO_2_ is widely used as a flocculent, disperser, and whitening agent in the paints and coatings industry. TiO_2_ along with other colored pigments is used in several end-user products such as emulsion paints, automotive coatings, aircraft coatings, etc. In automotive varnishes, the manufactured good is employed as a dispersive agent in conjunction with the highest gloss retention and elevated chalk resistance. These elements contributing to the automotive sector are supposed to have a definite impact on the global market over the projected period. Overall, according to the global market report, TiO_2_ market is expected to increase from USD 15,405.5 million in 2017 to USD 20,530.1 million by 2024, with a compound annual growth rate (CAGR) of 4.2% [5]. Annually about 4 million tons of TiO_2_ produced globally, and about 3000 tons of that process in the nano-scale form [6]. Since 2005, the number of products containing NPs available in commercial markets increased from 54 to 2850 in 2016. According to nano-databases, TiO_2_ NPs are used in about 25% of the products including paints [7], pigments, cosmetics, food processing and packaging (under E code number E171 as a food colorant), nano-fertilizers or nano-pesticides, biomedicine and clean-energy appliances like solar cells and also as part of pollutant removal from wastewater [8].

**Figure 1:**
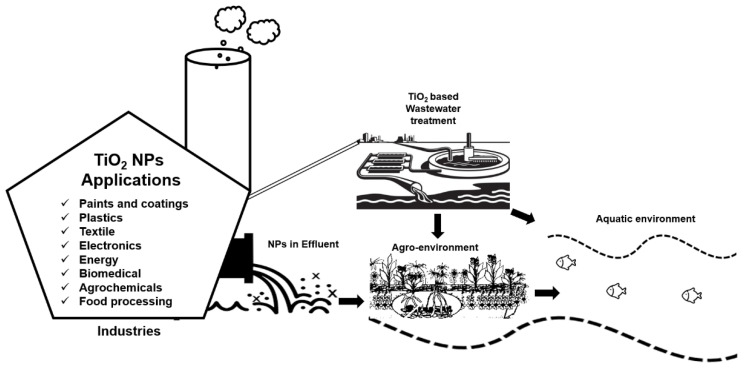
Illustration of the wide range of TiO_2_ NPs applications from industries, their release into the wastewater, and their possible exposure routes towards the agro-environment.

TiO_2_ NPs are one of the most extensively used NPs in different sectors [9]. For example, TiO_2_ NPs are widely used in the agriculture sector for different purposes such as nano-pesticides and nano-fertilizers to introduce sustainable agricultural practices [10]. The availability of these nano-based agrochemicals in the market is expected to rise in near future [11]. Similarly, the use of TiO_2_ NPs has also gained the utmost importance in other fields, and eventually from different sources, the inevitable release of these NPs into the environment is obvious either through a direct or indirect route. For example, in 2008, the first evidence of TiO_2_ NPs leaching (3.5 × 10^7^ NPs per L) into the aquatic environment from facade paints was reported [12]. In 2011, TiO_2_ NPs were first detected in effluents of wastewater treatment plants, which were discharged into freshwater bodies where these NPs can cause unknown ecological risks [13]. TiO_2_ NPs have also been observed to detach from some textiles and paints due to washing or weathering and to run into wastewater treatment plants [14,15] and especially in sewage sludge reaching the approximate concentration of 2 g·kg^−1^ [16]. Sewage sludge is commonly employed as soil fertilizer in agriculture at the rate of approximately 3 tons per hectare (on a dry weight basis) annually [17,18,19], and become an ultimate source of TiO_2_ NPs dissemination in agricultural soils. However, the overall concentration of these NPs in the environment through direct exposure route will be much higher than the indirect release. Interestingly, in both soil and water medium, TiO_2_ NPs can be used for purification purposes due to their unique characteristics of photocatalysis in the presence of ultraviolet (UV) light [20,21]. Figure 1 below illustrates the brief overview of TiO_2_ NPs applications, their role in wastewater treatment and their impacts on agro-environment which we have focused on in this review.

Although, TiO_2_ NPs offer several benefits, their hidden release into the environment poses potential risks to the entire ecosystem. Increased production and consumption of these NPs indicate their uncontrolled release into the environment, which raises serious environmental concerns that need to be studied [22]. There are three main environmental compartments such as air, water, and soil that provide prospective routes for NPs entrance. Plants also offer a potential route for the transfer of NPs to the environment and ultimately pave the way for their bioaccumulation into the food chain. As the environmental exposure of TiO_2_ NPs is increasing, humans are also more susceptible to these NPs, and they can easily enter the human body via various routes like oral, inhalation, and dermal contact [23,24]. Common people may be exposed to these NPs via drinking water, food ingestion, medications, and dermal contact with consumer products containing NPs. There are three major possible exposure routes of TiO_2_ NPs such as occupational, consumer, and environmental exposure. The present review summarized the recent developments of TiO_2_ NPs in the field of wastewater treatment, and the agro-environment, their environmental impacts along with an overview of their possible exposure routes. As nanotechnology is still in its infancy, so it is timely to consider the potential future problems it could cause before large amounts of NMs/products reach the market, and inevitably reach the environment. In this way, we may prevent undesirable large-scale effects through proactive approaches. 

## 2. Wastewater Treatments

**Figure 2:**
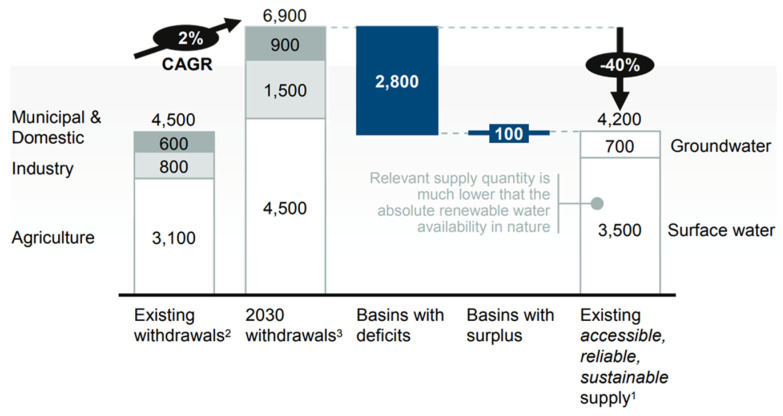
Comparison of current and future water demand, Reproduced with permission from [25], published by McKinsey & Company, New York, NY, USA, 2009.

With the onset of industrialization, there has been a steady increase in the types and amount of pollutants released in the environment. These environmental problems have garnered much attention on the global scale, especially water scarcity. Global water scarcity is a temporal and graphical mismatch between freshwater resources and the world’s water demand. The increasing world population and urban industrialization have made water scarcity more alarming as shown in Figure 2, predicting the gap between supply (4200 billion m^3^) and demand (6900 billion m^3^) of freshwater in 2030. A major proportion of this water is used for the agriculture sector and then for the industrial sector. 

With a growing world population, an ever-increasing demand for food production and potable water is questionable. The agriculture sector requires a surplus amount of water for irrigation. To avoid water scarcity issues, the reuse of wastewater is tremendously increasing across the planet. Reusing wastewater is a sustainable strategy to manage natural water resources [26]. However, the use of untreated wastewater for irrigation is a usual practice in developing countries causing serious threats to the ecosystem as well as human health. Specifically, carcinogenic pollutants pose a solemn threat to agricultural land, irrigated with industrial effluent without any treatment [27].

The whole world stands as a witness to unintended repercussions caused by rapid industrialization. The wastewater generated from industrial sectors has pronounced effects on humans as well as landmass fertility. Some industrial estates have operational wastewater treatment plants but unfortunately, they cannot handle a large proportion of industrial effluent. To meet the international standards of wastewater discharge, suitable technologies are required for wastewater treatment before discharging to streams. It could help to reduce the burden on freshwater resources by reusing treated water in various industrial processes. Due to the widely used application of nanotechnology, challenges, and opportunities of using engineered nanomaterials (ENMs) in wastewater treatment is a matter of endless concern. Based on the wastewater standards, a technique using TiO_2_ NPs for resilient pollutants in the context of wastewater treatment has become popular in recent years. Up to date, TiO_2_ NPs have drawn attention over other photocatalysts in every field of life. Over the last few decades, TiO_2_ NPs with high photocatalytic efficacy has been tested to reduce the pollution load from various industrial units. The conventional wastewater treatment methods mostly come up with high costs as well as lower efficiencies. However, the advantages of the use of TiO_2_ NPs (non-toxic, inexpensive, stable, and reusable NPs) appeared as a promising strategy to save the environment from pollution.

### 2.1. Slurry-Based Titanium Dioxide (TiO_2_) System

To date, a widely used photocatalyst in wastewater treatment is Degussa-P25, a trademark used for commercial TiO_2_ NPs. Very fine NPs of P25 TiO_2_ have been used in the form of slurry as reference material for comparison of photocatalytic degradation under various conditions. This is because in slurry form, these commercial NPs are always linked with volumetric production of reactive oxygen species (ROS) relative to active surface sites. TiO_2_ NPs have also played their role in the treatment of high strength industrial effluent (containing toxic organic and chlorinated compounds) generating from paper and pulp industries. In the pulp industry, the biodegradability index of effluent is 0.02–0.07 during bleaching of pulp which requires further treatment of the biological process for complete removal of these persistent pollutants [28]. Later in a study, where wastewater treatment was carried out using TiO_2_ NPs, the biodegradability index increased from 0.16 to 0.35 indicating the use of TiO_2_ NPs as an efficient pretreatment process before biological treatment step [29]. Some of the recent applications of TiO_2_ NPs in the form of a slurry, for wastewater treatment, are listed in Table 1.

### 2.2. TiO_2_-Based Photocatalytic Reactors

One of the essential aspects after the use of slurry-based TiO_2_ NPs is regeneration, which is an important concern for the case of economically viable water-treatment technology. The regeneration capability of nanomaterials might be reflected as an additional benefit for their attractiveness in water-treatment technologies. Several techniques have been used to resolve the problem associated with the additional cost of separating NPs from water including immobilization of NMs on adequate substrates and the use of different separation methods. Regeneration of NPs can be achieved efficiently using various photocatalytic reactors. Photocatalytic reactors are classified into two main configurations based on the deployed state of TiO_2_ NPs: (i) use of NPs in form of suspension and (ii) immobilization of NPs on inert carrier [40]. Downstream separation is required in the first type of configurations as compared to the other one which is a continuous operation. The various types of photoreactors, catalyst employed, and their mode of application are described in Table 2.

### 2.3. TiO_2_-Based Electrospun Nanofibers

Nowadays, TiO_2_ assisted photoreactors have become unfavorable, owing to the proper configuration and artificial light source which is associated with surplus use of electric power as well as treatment costs. The limitations associated with the use of TiO_2_ NPs in conventional ways paved a path for the synthesis of TiO_2_-based nanofibers (a one-dimensional form of nanomaterial) by electrospinning. One dimensional nanofibers are superior to NPs owing to intriguing characteristics such as; excellent charge carrier mobility, larger surface area, electrode availability to hole-transporting materials due to pores, improved charge collection as well as transport, and capability to assemble as free stand-alone membrane [51]. They can be synthesized in the form of thin mats and films with a fixed substrate with no need to recover the NPs after treatment.

With the entry of nanotechnology in every field of science, functional NPs can be easily immobilized/impregnated into polymer matrix for avoiding the costly downstream separation step. Furthermore, it also offers an opportunity to fetch priority contaminants that come close to the photocatalytic active sites for efficient utilization of short-lived reactive oxygen species (ROS), commonly known as “bait-hook and destroy strategy” [52]. Polymeric nanofibers can serve as a competent carrier of photocatalytic NPs for efficient industrial wastewater treatment. Photocatalytic degradation of organic contaminants using nanofibers has garnered much attention in recent years.

In the past few decades, several researchers have been devoted to the fabrication and characterization of electrospun TiO_2_ nanofibers where the precursor solution (polymeric solution) contains amorphous TiO_2_ followed by calcination at 500 °C. After calcination, obtained TiO_2_ nanofibers are transformed into crystallized forms (rutile and anatase) for efficient photocatalytic activity. TiO_2_ nanofibers were also synthesized using titanium-tetraisopropoxide (TTIP) and tetrabutyl titanate (Ti(OBu)_4_) as TiO_2_ precursors [53,54]. The commercial-grade TiO_2_ NPs Degussa (P25) has been used directly with a polymer blend for the fabrication of TiO_2_ nanofibers which did not require a calcination step afterward [55]. TiO_2_ NPs can easily be immobilized/supported on polymer nanofibers (either directly in polymer solution or decorated on the surface of nanofibers) with the advantage of efficient recovery after complete mineralization of pollutants [56,57]. Some of the studies on industrial wastewater treatment using TiO_2_ nanofibers are summarized in Table 3.

Heterogeneous photocatalytic degradation using TiO_2_ NPs has gained popularity as an effective alternative environment-friendly water treatment approach for a variety of water pollutants including organic and inorganic impurities in industrial effluent. Besides the tremendous use of TiO_2_ NPs in wastewater treatment, the inhibitory and biocidal effects of these NPs have been well known. In this context, they have exhibited an excellent broad-spectrum antibacterial activity against various microorganisms especially the pathogenic bacteria [62]. Before discharging industrial effluent into the agro-environment, TiO_2_ NPs-based treatment technique can be used that could help to completely mineralize water contaminants and eradicate the major concerns of the industrial wastewater treatment. Subsequently, the use of TiO_2_ NPs is highly anticipated for future studies owing to their effective photocatalytic property, and photo-stability. According to a modeling approach, among the ENMs released from wastewater treatment plants; the concentrations of TiO_2_ NPs in biosolids constitute about 263–367 mg kg^−1^, 273–342 mg kg^−1^, and 70–120 mg kg^−1^ in London, New York City, and Shanghai [63]. The biosolids produced during wastewater treatment are utilized as fertilizers in agriculture [64]. The application of biosolids to agricultural land leads to an increase in the release of TiO_2_ NPs in the soil [65]. In this context, the potential impacts, fate, and behavior of these NPs need to be investigated in the agro-environment.

## 3. Impacts of TiO_2_ Nanoparticles (NPs) in the Agro-Environment

**Figure 3:**
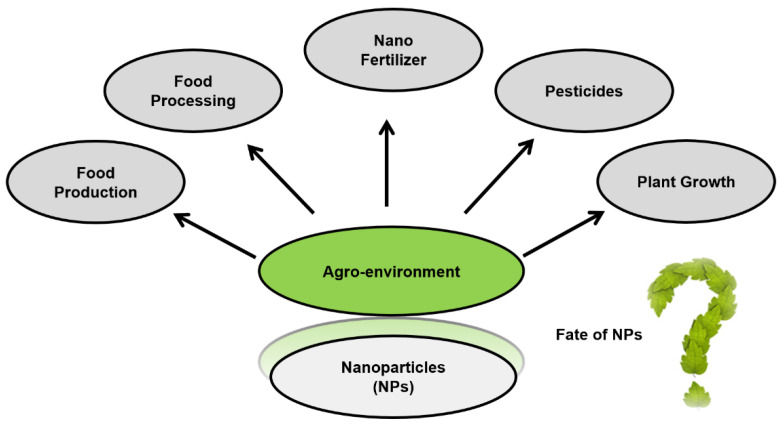
Applications of NPs in agro-environments.

In agro-environment, soil is the main and complex matrix in which analyzing the fate of TiO_2_ NPs is a challenging task. Furthermore, the impacts of TiO_2_ NPs are difficult to measure in the soil due to the high geogenic background of Ti (≈0.6% of the terrestrial crust). Up until now, modeling studies had helped to estimate the approximate amount of TiO_2_ NPs that is accumulating in the environment. According to recent forecasts, TiO_2_ NPs sludge treated soils (with 45,000 tons) were observed to be the largest sink for NPs release among different environmental compartments [16]. The crop plants served as an entry route for NPs’ uptake into the food chain. Presently, there are limited data available about these NPs interactions within the soil matrix. As nanotechnology is emerging in the field of agriculture sector in terms of growing global food production, nutritional contents, quality, food safety, and security [66]. Besides all these aspects, there are several other applications of NPs in agro-environments as shown in Figure 3, such as food processing and production, nano-fertilizer, nano-pesticides, etc. but the important concern arises here is the fate of these NPs.

**Figure 4:**
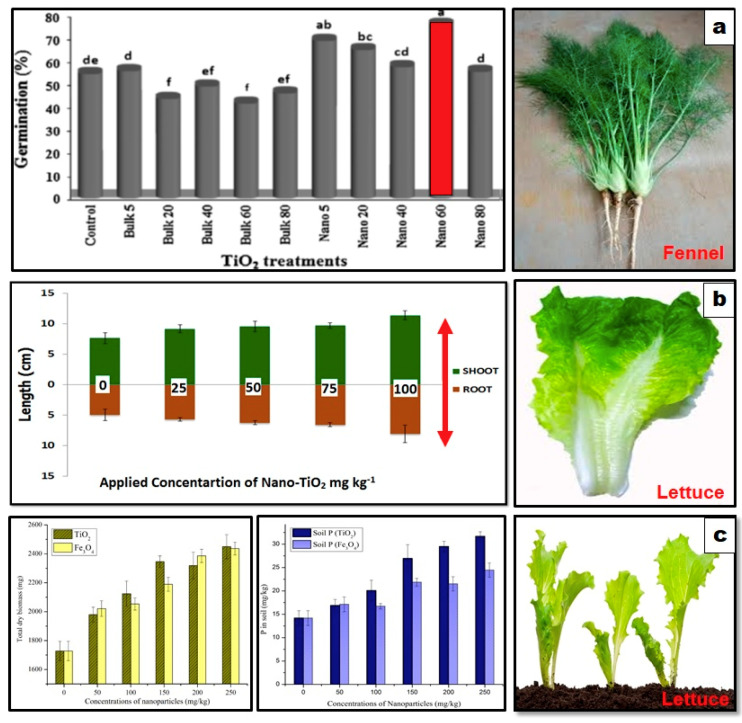
Effects of TiO_2_ NPs on plants with respect to different stages, concentration range, and exposure time. (**a**) represents the effects of TiO_2_ NPs on germination % of fennel seeds after short term exposure in a petri dish, the lowercase letters show the level of significance such as ‘a’ represent significant increase in germination percentage at Nano 60 treatment compared to control group. Adapted with permission from [71], published by ELSEVIER, 2013, (**b**) shows the effects of TiO_2_ NPs on plant length after short-term exposure in soil Adapted with permission from [72], published by Society for the Advancement of Agricultural Sciences Pakistan, 2015, (**c**) shows the effects of these NPs on lettuce plants after long term exposure of 90 days in soil, Adapted with permission from [73], published by American Chemical Society, 2015.

Scientists have investigated the effects of TiO_2_ NPs on the soil–plant continuum and have observed diverse impacts based on different characteristics of NPs, plant species, experimental conditions, and exposure period. For example, Figure 4, shows the TiO_2_ NPs effects on plants with respect to different stages, concentration range, and exposure time. In a recent study, experiments were conducted on growth-promoting rhizobacteria (PGPR) inoculation with and without TiO_2_ NPs in peat soil under the three stress situations. TiO_2_ NPs were reported to enhance the performance of growth-promoting rhizobacteria which further promotes the solubilization of insoluble phosphates [67]. A grassland soil was treated with TiO_2_ NPs at the rate of 0, 500, 1000, and 2000 mg kg^−1^ of soil. These NPs were observed to negatively affect the soil bacterial communities after 60 days of exposure [68]. TiO_2_ NPs effects on several bacterial taxa were also studied using incubated soil microcosms having concentrations range of TiO_2_ NPs 0, 0.5, 1.0, and 2.0 mg g^−1^ soil. Of the identified taxa that exist in all samples, 9 taxa were found to be positively correlated with TiO_2_ NPs, 25 taxa were negatively correlated whereas 135 taxa were not affected by TiO_2_ NPs [69]. In another study, TiO_2_ NPs effects were investigated at concentrations ranging from 0.05 to 500 mg kg^−1^ dry soil on different bacterial communities. The abundance of ammonia-oxidizing archaea was reported to decrease by 40% in response to TiO_2_ NPs whereas *Nitrospira* was not affected at all. Furthermore, the abundance of ammonia-oxidizing bacteria and *Nitrobacter* were also reported to reduce due to TiO_2_ NPs treatments [70].

TiO_2_ NPs (0, 5, 20, 40, 60, and 80 mg/kg) were used to study phytotoxicity and stimulatory impacts on fennel after 14 days of exposure. The mean germination percentage was increased by 76% at 60 mg L^−1^, while the mean germination time was decreased by 31% at 40 mg L^−1^ [71]. Similarly, in another study, plant shoot-root length was increased by 49% and 62%, respectively at 100 mg kg^−1^ of NPs treatment in lettuce after 14 days exposure in soil medium [72]. Another study was performed using TiO_2_ NPs treatments (0, 50–250 mg kg^−1^) in soil medium for a period of 90 days. The total dry biomass was observed to increase 1.4-fold and phyto-available phosphorus (P) in soil by 2.2-fold, respectively [73]. Table 4 enlists the recent studies conducted for the investigation of TiO_2_ NPs effects on different plants.

Studies have shown the positive effects of TiO_2_ NPs on the physiology of red bean plants, leaving no negative biochemical impacts in plants [97]. Low concentrations of TiO_2_ NPs were reported with their positive effects on chickpea cells especially when they were exposed to cold stress. However, TiO_2_ NPs especially at 5 mg kg^−1^ concentration level was reported to reduce cold-induced damages in sensitive and resistant chickpea genotypes. Such domino effects raised key questions regarding the potential mechanisms. It was supposed that the activation of the defensive mechanisms in chickpea seedlings after the absorption of TiO_2_ NPs support the plants in cold stress. These results are quite interesting for further practice in cases of environmentally stressed conditions. These new findings could pave the way to increase the use of NPs especially to improve the cold stress tolerance in major crops [98]. Furthermore, in future studies, TiO_2_ NPs application in combination with fertilizers could be an effective option to search out a way for better application of these agrochemicals in a sustainable way. We further need to explore the potential of nanotechnology by upscaling the present studies by investigating the effects of NPs at different stages in the life cycle of plant species and understand their mechanism of environmental exposure.

## 4. Understanding the Mechanism of Environmental Exposure of NPs

**Figure 5:**
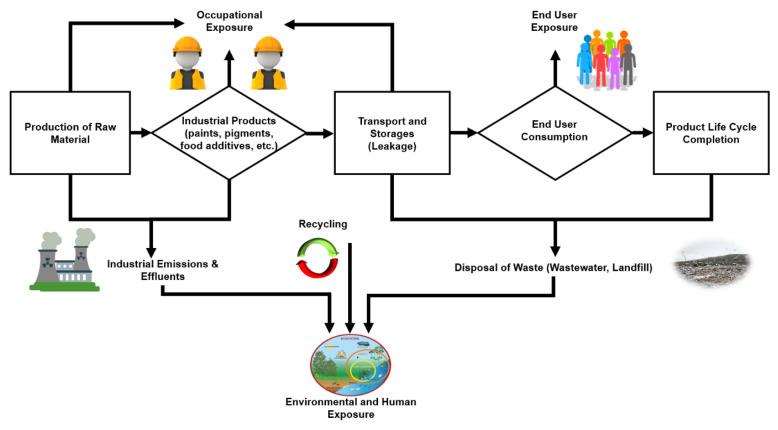
Probable routes of human and environmental exposure.

The increased use of NPs in different fields has raised a worldwide concern regarding their release and impact on human health and the environment. For this reason, in the recent decade, toxicological effects of NPs on human health and the environment also gained attention. The potential for exposure to these NPs begins with the production of these materials until their associated life cycle completion and release into the air, soil, and water [99] as shown in Figure 5.

Among possible exposure routes of TiO_2_ NPs, there are three major exposure routes including occupational exposure, consumer exposure and environmental exposure.

### 4.1. Occupational Exposure via Industries

According to a Swiss survey report, the usage of TiO_2_ NPs increased in amounts of approximately more than 1000 kg per company annually [100]. Occupational exposure to NPs may occur through dermal contact and dust inhalation at workplaces or industries; where these NPs are used or manufactured. The National Institute for Occupational Safety and Health (NIOSH) reported that the workers over the industrial units are at high risk of exposure to NPs due to unintentional hand-to-mouth touch [101]. Usually, the materials at microscale levels are considered to be harmless, however recent studies suggested that frequent inhalation of NPs could be dangerous [102]. The impact of NPs on humans has been investigated using various rodent models through various exposure routes and conditions. For example, inhalation of TiO_2_ NPs was reported to cause lung damage in mice due to inflammation, pulmonary fibrosis, and initiation of lung tumors [103]. In the human body, the liver is the most susceptible organ targeted by NPs [104]. TiO_2_ NPs have been reported to induce toxic effects on the liver affecting its functions [105]. In China, a study regarding occupational exposure to TiO_2_ NPs was conducted in a packaging workshop. Workers were selected on the basis of age (20 years or more) and employment (at least one year). Cardiopulmonary effects through possible biomarkers and physical experiments were conducted to reveal TiO_2_ NPs exposure. A pattern having time (dose)–response was observed in exposed workers, suggesting that long-term exposure to TiO_2_ NPs cause serious threats through occupational exposure [106].

TiO_2_ NPs have the ability to generate reactive oxidative species and oxidative stress even at lower NPs concentration. In a recent study, an acute exposure of TiO_2_ NPs to human lungs resulted in substantial modifications in gene expression along with long-term effects on progeny cells even after multiple generations via transcriptional changes [107]. Similarly, genotoxicity and cytotoxicity of TiO_2_ NPs (having different shapes) in bronchial epithelial cells were studied. Genotoxicity was determined on the basis of cellular-uptake as well as the ability of NPs to aggregate, whereas lesser cytotoxicity of NPs was observed to be significantly influenced by irradiation time and the shape of TiO_2_ NPs [108]. Another study reported that in case of acute exposure conditions, TiO_2_ NPs did not cause cytotoxicity in human alveolar A549 cells [109], but there is a lack of information related to the magnitude of NPs released and exposed to organisms, which can be better interpreted in future studies [110].

### 4.2. Consumer Exposure 

Consumer products available in markets including i.e., cosmetics, beverages and food, appliances, health and fitness, gardens and homes, etc., contain NPs and are expected to have direct consumer exposure [111]. TiO_2_ NPs having different sizes were used to investigate their effects on a human keratinocyte cell line (HaCaT) and reported that all the tested types of TiO_2_ NPs increased the superoxide production, and apoptosis in a dose-dependent manner [112]. Another study reported that TiO_2_ NPs at the half maximal effective concentration (EC_50_) ranges from 10^−4^ to 10^−5^ mol L^−1^ induced cytotoxic effects on HaCaT cells [113]. Recently, in another study the authors investigated the TiO_2_ NPs in combination with the ingredients from modern lifestyle products like cosmetics, skin-care products, and Henna tattoos. TiO_2_ NPs alone were not reported to induce any damage in cell viability upon application of 100 µg mL^−1^ up to 24 h [114]. Recently a social survey was conducted in USA regarding the individual exposure to TiO_2_ NPs used in personal care products. From these results, toothpaste and sunscreen were considered as the major source of dermal exposure depending on their usage pattern and amount of TiO_2_ NPs in these products. It is estimated that a person can exposed to 2.8 to 21.4 mg TiO_2_ per day through dermal exposure. Per day oral exposure is estimated from 0.15 to 3.9 mg TiO_2_ via toothpaste [115].

Over the course of history, TiO_2_ has been considered to pose low toxicity both for humans and the environment. Since ancient times, it has been the most widely used material as a coloring agent [116]. However, in 2018 the French national assembly revised the guidelines with the amendment to ban the use, import, and sale of nano-scale TiO_2_ as a food additive in any kind of food by 2020 [117]. Because of the fact that limited information is available on the safe usage of these NPs in consumer products, their potential hazards for their users need to be assessed. There are several factors involved in the assessment of the consumer exposure to these NPs that constrained due to the limited access of information; (a) list of commercial products containing NPs, (b) amount of NPs used in such products, and (c) behavior of the consumer towards them [118]. Most of the commercial products containing these NPs do not enlist this information on their ingredient lists. Moreover, the number of consumers of such products and industry-derived data are kept hidden from all stakeholders including the governments, public, and private sectors which makes the consumer exposure situation more alarming [119].

### 4.3. Environmental Exposure 

The term environmental exposure is based on the extent of NPs taken up by biota, either in metabolized or degraded form, and their rates of excretion. This is where the least amount of data is available, and particularly data that consider the modifying effects of the environments where organisms live while they are exposed to NPs. In the product life cycle starting from manufacturing until consumer usage, each stage for NPs could result in their release into the environment. The tendency for physicochemical properties varies as these NPs move from different environmental compartments such as water, soil, and air. Understanding the importance, their fate, transport, and transformation need to be emphasized. However, little is known about what governs these processes for NPs in general. So, we tried to summarize what is known about the environmental behavior of these NPs which is as follows.

Upon release into the environment, NPs usually act in one or more of the following ways: (1) stay suspended as an individual particle; (2) form agglomerates (and potentially sorbed onto some surface or experience facilitated transport); (3) dissolve in a liquid; and (4) transform chemically by reacting with organic matter or other natural particles. The extent to which NPs’ behavior follows any of the aforementioned patterns depends on their surrounding environment, and several biological, chemical, and physical processes. Nanosize TiO_2_ made them extremely mobile in the soil system, but their larger surface areas (compared to their size) enhance their tendency to sorb onto the soil, which restricts their movement or makes them immobile. For example, TiO_2_ NPs considered as having low solubility, remained in the soil for long periods which might create potential environmental risks for deeper soil layers. Small-sized TiO_2_ NPs (20 nm) were able to penetrate the plant cell wall and have been reported to reduce wheat’s biomass [120]. Plants offered a potential route for the transmission of NPs to the environment and ultimately paved the way for their bioaccumulation into the food chain. Different studies have determined the response of NPs to plants growth and their possible mechanism. Plant cell walls do not allow the smooth entrance of any external agent as well as NPs into the plant cells. The screening property of the cell wall depends on the diameter of pores present in the cell wall that mostly ranges from 5 to 20 nm [121]. Therefore, NPs and their accumulates within the stated range could simply cross the cell membrane and transfer to the plant’s aerial parts. NPs might generate various morphological changes in the root structures, which increases pore sizes or generates new pores in the cell wall, which further enhances the uptake of NPs and their aggregates [122]. A recent study reported that TiO_2_ NPs from the environment undergoes the size selection process during the foliar and root uptake mechanism in *Dittrichia viscosa* wild plants. The study reported that the TiO_2_ NPs having a size less than 50 nm were accumulated in plant’s leaves (53%), stems (90%), and roots (88.5%) [123]. In another study, TiO_2_ NPs of size 4 and 150 nm were reported to be internalized through foliar uptake in lettuce plant leaves via stomata [124]. Another report stated that NPs accumulated on photosynthetic surface-induced foliar heating that can alter the gaseous exchange due to stomatal disturbance. Consequently, altering the different molecular and physiological functions of plants [125]. Therefore, the influence and translocation of different NPs within plants need to be investigated further to understand the whole mechanism and their behavior in plants [126]. As the human food chain instigated with plants, so it is critically important to understand how plants respond differently to these NPs which are frequently concentrating in our ecosystem through various routes.

Aquatic systems are usually considered as the main recipient of NPs. As in the terrestrial environment, transformation in the aquatic system includes several, physical (aggregation/agglomeration and deposition), biological (interaction with macromolecules including polysaccharides, proteins, and surfactants) and chemical processes (dissolution, sorption, and redox reactions). Aside from the intrinsic properties, transformation, and toxicity of TiO_2_ NPs also rely on various environmental factors such as temperature, pH, light, and presence of natural organic matter [127]. In natural aquatic systems, many organisms are sensitive to NPs’ exposure and exhibit pronounced toxic effects during their transport and transformation. This might be because NPs have surface coatings that help to improve their solubility and suspension and made them more mobile than the other large-sized particles. TiO_2_ NPs have been reported to induce a significant decrease in growth parameters of an aquatic plant *Spirodela polyrrhiza*, whereas the increased concentration of TiO_2_ NPs was observed to increase the photosynthetic pigmentation and the peroxidase activity [128]. Overall, the negative effects of nanoparticles must not be ignored, especially on human health and the environment, and must be studied in detail to make their use controlled and safe. Table 5 below briefly enlists some studies of TiO_2_ NPs on terrestrial and aquatic organisms.

## 5. Conclusions and Future Perspectives

This review briefly discussed the recent developments of TiO_2_ NPs in wastewater treatment technologies and the agro-environment. The potential exposure pathways of these NPs and their associated environmental risks were also highlighted. In fact, the use of TiO_2_ NPs will further increase for promising applications in the near future. In wastewater treatment technologies, downstream separation of these NPs after photocatalytic degradation is still a matter of concern which can be minimized by using TiO_2_ in photocatalytic reactors either in slurry form or immobilized on a solid substrate. Immobilization might result in loss of potential active sites which could be minimized by adding NMs into the polymeric substrate. The polymer can provide firm anchoring to TiO_2_ NPs, however, there is still a chance of NPs leaching into the treated water and reaching the agricultural soils via irrigation. Since the agriculture sector is the backbone of the economy in most countries, studies based on crop improvement using TiO_2_ NPs could help to overcome the burden of nutrient deficit in soils providing better crop yield. Apart from the potential benefits of TiO_2_ NPs there are also some limitations that we could not ignore. At this stage, we could not claim with surety that the use of NPs is fully safe for human health and the environment or if it is harmful. Risks associated with chronic exposure of these NPs, interaction with flora and fauna, and their possible bioaccumulation effects have not been fully considered yet. The other limitations include the lack of information about a safe range of NPs’ concentration, scalability of research and development for prototypes, industrial production, and public concern about health and safety issues. Detailed investigations are necessarily required to resolve these concerns and provide conclusive statements. We need to optimize the useful concentration levels of TiO_2_ NPs for various applications and limit their usage for environmental safety.

## Figures and Tables

**Table 1:** TiO_2_ NPs applications for photocatalytic degradation of industrial wastewater treatment.

Type of Pollutant	Photo-Catalyst	Experimental Conditions		Light Source	Photocatalytic Activity	Ref.
		Catalyst Dose	Contaminant Conc.			
Dimethyl arsenic acid (DMA)	Mesoporous TiO_2_ NPs	0.8 g/L	200 µg/L, 100 mL	300 W Xe-arc lamp	95.12% DMA removal at pH 7.5 and further increase was observed between pH 3–5	[30]
Methylene blue (MB) and Congo red (CR)	TiO_2_ NPs	25 mg/mL	15 mL, MB (10 mg/L), CR (20 mg/L)	UV–Vis light (λ = 304–785 nm)	85% MB removal at pH 11.25, 99.7% CR removal at pH 5.40	[31]
Chemical Oxygen Demand (COD) and SO_4_^2−^ from oil refinery wastewater	TiO_2_ NPs	0.5–1.5 g/L	1 L real refinery effluent	18 W UV lamp (λ = 400 nm)	91.21% of COD and 86% SO_4_^2−^ removal after 15 min	[32]
Rhodamine B	Porous TiO_2_ NPs	0.100 g/100 mL	400 mg/L	300 W tungsten filament solar lamp	98% degradation within 20 min	[33]
Refinery wastewater	TiO_2_ NPs	100 mg/L	150 mL real refinery effluent	6 W low-pressure mercury vapor lamp (λ = 254 nm)	32% Total Organic Carbon (TOC) and 67% Total Nitrogen (TN) after 90 min	[34]
Tannery wastewater	TiO_2_ NPs	5 g/L	5 L real tannery effluent	Solar radiations of intensity 985 W/m^2^	83% COD and 76% Cr^+6^ after 5 h	[35]
Rhodamine B	TiO_2_ NPs	20 g/L	4 mg/L, 400 mL	Visible light (λ ~ 365 nm)	65% Rhodamine B degradation	[36]
1,4-dioxane	Degussa P25-TiO_2_	1.24 g/L	(25, 50, 100, 150 and 200 mg L^−1^), 20 mL	1000 Wm^−2^ Xe lamp (λ = 315–400 nm)	50% COD and 40% TOC after 6 h	[37]
Rhodamine B	Degussa P-25 TiO_2_	1.6 g/L	20 mg/L, 25 mL	Blue UV light (λ = 390–410 nm)	96% degradation in 60 min	[38]
Acid Orange 7	Degussa P-25 TiO_2_	0.5 g/L	40 mg/L, 800 mL	400 W HP Hg lamp (λ = 253.7 nm)	100% degradation in 120 min	[39]

**Table 2:** Overview of various types of reactors, the catalyst employed, and their application in wastewater treatment.

Reactor Type	Reactor Name with Photocatalyst	Target Pollutant Conc.	Findings	Ref.
Suspended	Baffled reactor using Degussa P25-TiO_2_ NPs	Acid orange 52 (50 mg/L)	Complete mineralization after 30 h at a flow rate of 14.4 L/h	[41]
	Submerged membrane photocatalysis reactor (SMPR) using UV/TiO_2_	Rhodamine B	95% removal was observed at a catalyst loading of 0.1 g/L under 3 ultraviolet (UV) c lamps at pH 8	[42]
	Slurry photoreactor having mesoporous TiO_2_ NPs	Dichlorophenol-indophenol (DCPIP) dye (1 to 4 × 10^−4^ mol/L)	96.4% DCPIP degradation occurred within 3 min at 1 × 10^−4^ mol/L concentration and pH 3	[43]
	Packed bed photoreactor	Phenazopyridine (10, 20, 30, 40 mg/L)	90% decrease in TOC was observed after 150 min	[44]
	Photocatalytic drum reactor having TiO_2_ NPs	MB (10 μM) and 4-Chlorophenol (100 μM)	93% MB degradation after 15 min and 94% 4-CP removal after 90 min	[45]
	Rotating drum reactor having Degussa P25-TiO_2_	MB	98% of MB removal was observed at 30 g/L TiO_2_ after 60 min	[46]
Immobilized	Thin-film fixed bed reactor having TiO_2_ NPs	Carmoisine dye (10 mg/L)	97% removal was observed at pH 2 after 45 min at a flow rate of 0.25 L min^−1^	[47]
	Baffled reactor immobilized with TiO_2_ NPs	Acid orange 52 (AO52) (50 mg L^−1^)	After 4 h, dye converted into benzene annular compound, intermediates gradually decreased after 10 h and complete mineralization into CO_2_ and H_2_O in 30 h	[41]
	Rotating disc photoreactor, TiO_2_ (P25) immobilized on High Density Polyethylene (HDPE) plate	p-nitrophenol (15 mg L^−1^)	83% removal was observed at pH 5 after 118 min at 800 mL volume	[48]
	Rotating aluminum drum with TiO_2_-coated corrugated aluminum drum	Tetracycline (0.5, 1, 5, 10, 30, 50, 60, and 80 ppm)	93% Tetracycline was observed after 20 min	[49]
	Spiral photoreactor system sintered with TiO_2_ thin film	4-tert-octylphenol (4-t-OP) (2, 5, 8 and 10 mg L^−1^)	90% 4-t-OP degradation was observed at 10 mg/L concentration with single layer TiO_2_ film (13.6% TiO_2_ precursor)	[50]

**Table 3:** Overview of various types of pollutants and TiO_2_ based nanofiber photocatalysts employed for wastewater treatment.

Type of Pollutant	Photo-Catalyst	Experimental Conditions		Light Source	Photocatalytic Activity	Ref.
		Catalyst Dose	Contaminant Conc.			
MB	Carbonized TiO_2_ nanofibers	2, 4 and 6 mg/40 mL	MB-blue (10 mg/L)	300 W Xenon lamp	At 4 mg dose, 94.98 ± 0.02% degradation was observed after 120 min which decreased up to 83.20 ± 0.01% after 5th cycle	[58]
MB	TiO_2_ NPs supported on Polyethylene terephthalate (PET) nanofibers	0.0032 g of TiO_2_ adsorbed on 0.011 g of PET nanofibers in 10 mL	MB (10 mg/L)	100 W Xenon lamp	88% degradation after 10 min	[57]
MB, Bisphenol A (BPA) and 17α-ethynylestradiol (EE2)	TiO_2_ nanofibers	4 × 5 cm^2^ rectangular coupons/50 mL	MB (6.4 mg/L), BPA and EE2 (C_0_ = 5.0 mg/L)	Six UV-A lamps (λ = 365 nm)	97% MB adsorbed in 240 min and degraded completely in less than 90 min, 96% removal for BPA and EE2 within 4 h and 1.5 h, respectively	[56]
MB	Polymethyl methacrylate (PMMA)/TiO_2_ nanofibers	3 × 3 cm^2^ rectangular coupons/50 mL	MB (10 mg/L)	8 W UV (λ = 254 nm)	20% degradation after 180 min	[59]
Rhodamine B	TiO_2_ nanofibers	0.1 g/100 mL	Rhodamine B (5 mg/L)	500-Watt tungsten halogen lamp (λ ~ 420 nm)	99% of degradation was observed after 2.5 h for nanofibers calcined at 500 °C	[60]
CR	Porous TiO_2_ nanofibers after silica leaching	0.5 g/L	CR (20 mg/L)	UV irradiation in a photochemical reactor	76.56 wt% photocatalytic degradation after 1 h	[61]

**Table 4:** TiO_2_ NPs applications since 2010 on different plants and their impacts.

Experimental Conditions	Plants	Impacts of TiO_2_	Ref.
TiO_2_ NPs Size: 20–30 nm Treatments: 0, 50, 100 and 200 mg L^−1^) in the growth medium of cocopite and perlite. Period: 60 days	Moldavian balm	Plants cultivated in salt stress conditions were observed to have improved physical traits and increased antioxidant enzyme activity in response to TiO_2_ NPs treatment compared to control.	[74]
TiO_2_ NPs Size: 50 and 68 nm Treatments: 100 mg *n*TiO_2_/kg on 10 mg kg^−1^ of Cd-spiked soils Period: 14 days	Cowpea	No change in chlorophylls occurred. In leaves and roots, both ascorbate peroxidase and catalase activities were improved by NPs. TiO_2_ NPs have the potential for soil nano-remediation and could be an environmentally friendly option to tolerate soil Cd toxicity in cowpea plants.	[75]
TiO_2_ NPs Size: 30 nm Treatments: 0, 30, 50 and 100 mg kg^−1^ Period: 60 days	Wheat	TiO_2_ NPs without P fertilizer increased Ca (316%), Cu (296%), Al (171%), and Mg (187%) contents in shoots at 50 mg kg^−1^ TiO_2_ NPs treatment which shows improved grain quality and crop growth.	[76]
TiO_2_ NPs Treatments: 0, 5, 10, 15, and 20 mg L^−1^ (foliar spray) Medium: Soil Period: 55 days	Rice *(Oryza sativa)*	The foliar spray of TiO_2_ NPs reduced the soil bioavailable Cd by 10, 14, 28, and 32% in response to 5, 10, 20, and 30 mg/L NPs treatments compared to their control values. These NPs also significantly decreased the Cd concentration in the shoot as well.	[77]
TiO_2_ NPs Size: <40 nm Treatments: 0, 50, and 100/mg kg^−1^ Medium: Soil Period: 40 days	Wheat *(Triticum aestivum)*	Shoots and root lengths of wheat plants increased by16% and 4%, respectively. Phosphorus in shoots and roots was increased by 23.4% and 17.9% at 50/mg kg^−1^ of soil compare to control.	[78]
TiO_2_ NPs Size: <40 nm Treatments: 0, 25, 50, 150, 250, 500, 750 and 1000 mg L^−1^ Medium: Soil	Wheat *(Triticum aestivum)*	TiO_2_ NPs at the highest treatment level of 1000 mg kg^−1^, plant growth, biomass. Phosphorus content along with other tested parameters did not shown any improvement in the testing soils.	[79]
TiO_2_ NPs Treatments: 0, 100 and 500 mg kg^−1^ Medium: soil Period: 60 days	Wheat *(Triticum aestivum)*	No effect of phytotoxicity was observed in plant growth, chlorophyll content, and biomass.	[80]
TiO_2_ NPs Treatments: 0–750 mg kg^−1^ Medium: Soil Period: 90 days	Rice *(Oryza sativa)*	Phosphorus concentration was increased in roots by 2.6-fold, shoots 2.4-fold, and grains 1.3-fold upon 750 mg kg^−1^ of NPs treatment. Metabolomics study revealed that levels of amino acids, glycerol content, and palmitic acid were also improved in grains.	[81]
TiO_2_ NPs Treatments: 0, 100, 150, 200, 400, 600, and 1000 mg L^−1^ Medium: Hydroponics Period: 7 days	Barley (*Hordeum vulgare* L.)	No adverse effect on shoot growth. Root growth inhibited as the concentration of TiO_2_ NPs increases. No effect on chlorophyll *a* and *b*. No significant effect on biomass.	[82]
TiO_2_ NPs Treatments: 0–100 mg kg^−1^ Medium: Soil Period: 60 days	Wheat *(Triticum aestivum)*	NPs treatment at the rate of 20, 40, and 60 mg kg^−1^ increased plant growth and phosphorus uptake. 32.3% of chlorophyll content increased at 60 mg kg^−1^ while 11.1% decrease at 100 mg kg^−1^.	[83]
TiO_2_ NPs Size: >20 nm Treatments: 0, 100, 250, 500 and 1000 mg L^−1^ Medium: Soil Period: 5 weeks	*Arabidopsis thaliana* (L.)	Plant biomass and chlorophyll content decreased as the NPs treatment increase. Higher concentrations of NPs improved root growth. NPs treatments from 100 to 1000 µg mL^−1^ affect vitamin E content in plants. Decrease in plant biomass by 3-fold in response to 500 and 1000 mg/mL NPs treatment, whereas, at 100 mg/mL, the biomass decreases to half relative to control.	[84]
TiO_2_ NPs Treatments: 250 and 500 µg/mL	Cabbage, Cucumber, Onion	The germination of cabbage significantly increased. In cucumber and onion, significant root elongation was observed.	[85]
TiO_2_ NPs P25: 29 ± 9 nm, E171: 92 ± 31nm, Non-nanomaterial TiO_2_: 145 ± 46 nm Treatments: 1, 10, 100, 1000 mg kg^−1^ Period: 12 weeks	Wheat, Red clover	TiO_2_ NPs showed restricted mobility from soil to leachate. No significant translocation of Ti was observed in both plant species, while average Ti content increased from 4 to 8 mg kg^−1^ at the highest treatments.	[86]
TiO_2_ NPs Size: 22 and 25 nm Period: 6 weeks	Soya bean	Plant growth significantly decreased which corresponds to the reduced carbon content in leaves.	[87]
TiO_2_ NPs Treatments: 0, 10, 20, 40 and 80 mg L^−1^ Medium: Petri dish Period: 10 days	*Alyssum homolocarpum, Salvia mirzayanii, Carum copticum, Sinapis alba*, and *Nigella sativa*	TiO_2_ NPs affected the germination and seedling vigor of 5 medicinal plants. Appropriate concentration levels had improved the germination as well as the vigor index of the subjected plant.	[88]
TiO_2_ NPs Treatments: 0, 10, 20, 30, and 40 mg mL^−1^	Parsley	Significant increase in seedlings germination percentage, germination rate index, shoot-root length, fresh biomass, vigor index, and chlorophyll content. 30 mg mL^−1^ was observed to be the optimum concentration of NPs. Increased germination percentage (92.46%) was observed at 40 mg mL^−1^ treatment, relative to the lowest one (44.97%) at control.	[89]
TiO_2_ NPs Treatments: 0, 0.01%, 0.02%, and 0.03% Medium: Soil Period: 14 days	Wheat *(Triticum aestivum)*	Under the water-stressed conditions, the plant’s length, biomass, and seed number along with the other tested traits like gluten and starch content were increased at 0.02% of NPs treatment.	[90]
TiO_2_ NPs Size: 14–655 nm	Wheat *(Triticum aestivum)*	NPs treatment improved root length. NPs above 140 nm diameter are not accumulated in wheat roots. NPs above 36 nm threshold diameter, can be accumulated (at concentration 109 mg Ti/kg dry weight) in wheat root parenchyma cells but are unable to translocate to the shoot. Enhanced wheat root elongation was observed when exposed to 14 and 22 nm TiO_2_ NPs.	[91]
TiO_2_ NPs Size: 5 nm Treatments: 0.25% NPs Medium: Hoagland nutritive fluid Period: 35 days	*Arabidopsis thaliana*	Improved photosynthesis and growth in plants were reported. Generally, the absorption of light in chloroplast and light-harvesting complex II was supposed to be stimulated by TiO_2_ NPs; thus, enhancing the transformation of light energy to electronic energy, the evolution of oxygen, and water photolysis.	[92]
TiO_2_ NPs (43%) with sucrose coating Size: >5 nm	*Arabidopsis thaliana*	Results revealed that small NPs entered plant cells and got accumulated in distinct subcellular locations.	[93]
TiO_2_ NPs Size: <100 nm Treatments: 0, 5, 10 and 20 mg L^−1^ Period: 20 days	*Zea mays* L.	TiO_2_ NPs treatment significantly reduced the shoot, root biomass, and chlorophyll contents of leaves in a dose-dependent manner. Whereas positive effects were reported on the N, P, K, Zn Mn, and Cu contents except for Fe.	[94]
TiO_2_ NPs Size: <100 nm Treatments: 15, 30, 60, 120 and 240 mg L^−1^ Period: at different time intervals up to a maximum of 82 days	*Vicia faba*	TiO_2_ NPs were reported to induce variations in a meiotic activity which results in an increased number of chromosomal abnormalities in the plant’s reproductive parts.	[95]
TiO_2_ NPs Size: <100 nm (tetragonal crystals), <10 nm (spherical shape) Treatments: 50 mg L^−1^ Period: 3 days	*Vicia faba* L.	Based on the characteristics of size and shape, TiO_2_ NPs can induce different levels of toxicity in terms of seed vigor index, aberration index and oxidative stress in plants.	[96]

**Table 5:** TiO_2_ NPs effects terrestrial and aquatic organisms.

Experimental Conditions	Organisms	Impacts of TiO_2_	Ref.
**Terrestrial Organisms**			
TiO_2_ NPs Size: 25 nm Treatments: 500 and 5000 mg kg^−1^ Period: up to 48 days	Nematodes (*C. elegans*)	Increased generation of intracellular reactive oxygen species. Toxicity reduced and the lifespan of survived nematodes increased in response to TiO_2_ NPs exposure.	[129]
TiO_2_ NPs Treatments: 0, 5, 50, and 500 mg kg^−1^ Period: 120 days	Earthworm (*Eisenia fetida*)	Lower glutathione/oxidized glutathione (GSH/GSSG) ratio and significant decrease in superoxide dismutase (SOD) activity was observed for 500 mg/kg TiO_2_ concentration.	[130]
TiO_2_ NPs Size: 50–100 nm Treatments: 0, 150 or 300 mg kg^−1^ of dry soil Period: 15, 30, 60 and 90 days	Bacterial community and *Eisenia fetida*	Unamended and earthworm—amended soil increased certain available bacterial groups such as *Firmicutes* and *Acetobacter* whereas decreased *Verrucomicrobia* and *Pedobacter* abundance.	[131]
TiO_2_ NPs (anatase) Treatments: 10, 50, and 100 nm Period: 2–3 months	Mice	Intestinal inflammation with lower body weight. Mice with removed gut microbiota did not show this phenomenon.	[132]
TiO_2_ NPs Size: 23 ± 6.8 nm Treatments: 0.5, 2.5, and 10 mg kg^−1^ Period: 2 h and 35 days	Sprague–Dawley rats	Persistent inflammation of lung and liver genotoxicity.	[133]
**Aquatic Organisms**			
TiO_2_ NPs Treatments: 0.25, 0.5, and 1.0 mg L^−1^ Period: 21 days	*Daphnia magna*	TiO_2_ with 20% rutile and 80% anatase had a highest mortality rate as compared to other crystalline forms.	[134]
Biosynthesized TiO_2_ NPs Size: 43–56 nm Treatments: 0, 2.5, 5, 10, 20, and 40 mg L^−1^	Zebrafish (*Danio rerio*)	Significant malformations such as tail curvature, egg coagulation, bend the spine and delayed hatching was observed at a concentration of 2.5 mg L^−1^ during 8 to 120 h post fertilized period.	[135]
TiO_2_ NPs Treatments: 25, 125, and 250/mg L^−1^ Period: 28/days	Red swamp crayfish (*Procambarus clarkia*)	The mortality rate was observed to be 0, 3.3, and 10% in response to 25, 125, and 250/mg L^−1^ of TiO_2_ NPs, respectively.	[136]
TiO_2_ NPs Treatments: 1.0 and 5.0 mg L^−1^ Period: 4 and 14 days	Nile tilapia (*Oreochromis niloticus*)	Acute exposure caused oxidative stress with a decrease in catalase (60%), superoxide dismutase (27%), and glutathione peroxidase (37%), while 14 days of exposure elevated the catalase (61%), glutathione-S-transferase (54%), glutathione peroxidase (32%), and glutathione reductase (93%).	[137]
TiO_2_ NPs Treatments: 500, 1000, 1500, and 2000 mg L^−1^ Period: 24 h	Brine shrimp (*Artemia salina*)	Mortality rate of 5, 20, 20, 53, and 57% was observed in response to 0, 500, 1000, 1500, and 2000 mg L^−1^ TiO_2_ NPs, respectively.	[138]
